# Effects of altered *N*-glycan structures of *Cryptococcus neoformans* mannoproteins, MP98 (Cda2) and MP84 (Cda3), on interaction with host cells

**DOI:** 10.1038/s41598-023-27422-9

**Published:** 2023-01-20

**Authors:** Su-Bin Lee, Catia Mota, Eun Jung Thak, Jungho Kim, Ye Ji Son, Doo-Byoung Oh, Hyun Ah Kang

**Affiliations:** 1 Department of Life Science, College of Natural Science, Chung-Ang University, Seoul, 156-756 South Korea; 2 Korea Research Institute of Bioscience and Biotechnology (KRIBB), Daejeon, 34141 South Korea; 3 Department of Biosystems and Bioengineering, KRIBB School, University of Science and Technology (UST), Daejeon, 34113 South Korea

**Keywords:** Glycosylation, Glycobiology

## Abstract

*Cryptococcus neoformans* is an opportunistic human fungal pathogen causing lethal meningoencephalitis. It has several cell wall mannoproteins (MPs) identified as immunoreactive antigens. To investigate the structure and function of *N*-glycans assembled on cryptococcal cell wall MPs in host cell interactions, we purified MP98 (Cda2) and MP84 (Cda3) expressed in wild-type (WT) and *N*-glycosylation-defective *alg3* mutant (*alg3*Δ) strains. HPLC and MALDI-TOF analysis of the MP proteins from the WT revealed protein-specific glycan structures with different extents of hypermannosylation and xylose/xylose phosphate addition. In *alg3*Δ, MP98 and MP84 had truncated core *N*-glycans, containing mostly five and seven mannoses (M5 and M7 forms), respectively. In vitro adhesion and uptake assays indicated that the altered core *N*-glycans did not affect adhesion affinities to host cells although the capacity to induce the immune response of bone-marrow derived dendritic cells (BMDCs) decreased. Intriguingly, the removal of all *N*-glycosylation sites on MP84 increased adhesion to host cells and enhanced the induction of cytokine secretion from BMDCs compared with that on MP84 carrying WT *N*-glycans. Therefore, the structure-dependent effects of *N*-glycans suggested their complex roles in modulating the interaction of MPs with host cells to avoid nonspecific adherence to host cells and host immune response hyperactivation.

## Introduction

*Cryptococcus neoformans* is an encapsulated basidiomycetous yeast and opportunistic human pathogen. It is the main causative agent of fungal meningoencephalitis in immunodeficient individuals, such as patients with HIV/AIDS or organ transplant patients^1,2^. Its spores or desiccated cells are inhaled through the host’s respiratory tract, reaching the host’s pulmonary alveoli in the lungs^3^. The adhesion of inhaled *C. neoformans* to host lung epithelial cells is enhanced by adhesion-related molecules, such as phospholipase B1 and mannoprotein (MP) 84^4,5^. These fungal cells are mostly eliminated by immune cells, such as alveolar macrophages, in immunocompetent people^6,7^. However, in immunodeficient patients, *C. neoformans* survives and proliferates through various mechanisms, including preventing phagocytosis through capsule induction or secretion of various molecules, and eluding macrophages via host cell lysis^8,9^. After fungal cells invade the immune system, free yeast cells or macrophage-engulfed cells are disseminated to different organs via blood circulation^10^ and cause adverse effects. For example, when *C. neoformans* reaches the central nervous system, lethal meningoencephalitis occurs^11,12^.

Protein glycosylation is one of the post-translational modifications involving acetylation, phosphorylation, and methylation, which modulate the activities and properties of proteins^13^. Glycosylation is required for endoplasmic reticulum homeostasis by affecting the folding, stability, and function of proteins^14,15^. Moreover, the glycosylation of fungal pathogens is important for their pathogenicity^16^. Highly mannosylated cell wall proteins are common in fungal species and involved in host cell interactions in several pathogenic fungi, such as *Candida glabrata* and *Aspergillus fumigatus*^17,18^. In *C. neoformans*, virulence is abolished in the *ktr3*Δ mutant strain, which has defective *O-*mannosylation^19^, and the *alg3*Δ strain, which generates truncated core *N*-glycans^20^. These results demonstrate the importance of glycans in the pathogenesis of *C. neoformans*.

In *C. neoformans*, MPs account for less than 1% of the cryptococcal capsule weight. Nevertheless, several MPs are immunogenic antigens of hosts^21^. Purified MPs from cultured supernatant promote the activation of T-cells and secretion of cytokines, including TNF-α, IL-12, IFN-γ, and IL-6, by monocytes^22–24^. MP98 and MP84, isolated as immunoreactive antigens that highly stimulate host CD4^+^ T- and B-cells, have a chitin deacetylase domain and a Ser/Thr-rich region^25–27^. Cytokine secretion is significantly enhanced by the synergy of MP98 and CpG oligodeoxynucleotides in murine dendritic cells^28^. Recombinant MP84 expressed in *Pichia pastoris* inhibits the adhesion of acapsular *C. neoformans* strain to lung epithelial A549 cells; therefore, MP84 mediates the adhesion of *C. neoformans* to lung epithelial cells^4,29^. The chitin deacetylase Cda family, including MP98 and MP84, is a major protein family typically found in *Cryptococcus* extracellular vesicles^30^.

In this study, to define the roles of *N*-glycans assembled on the cell surface MPs in *C. neoformans* pathogenicity, we investigated the effect of altered *N*-glycan structures on the interaction of cryptococcal MPs with host cells. We generated recombinant MP98 and MP84 that either carried the truncated core *N*-glycans or completely lacked *N*-glycans. We analyzed the *N*-glycan structures of MPs and examined their activities in adhering to host cells, uptake by dendritic cells, and inducing the cytokine secretion of host cells. Overall, our results revealed the potential structure-dependent roles of *N*-glycans in modulating the interaction of *Cryptococcus* MPs with host cells to avoid nonspecific interaction and hyperactivated immune responses.

## Results

### Secretory expression of MP98 and MP84 with truncated core *N-*glycans

**Figure 1:**
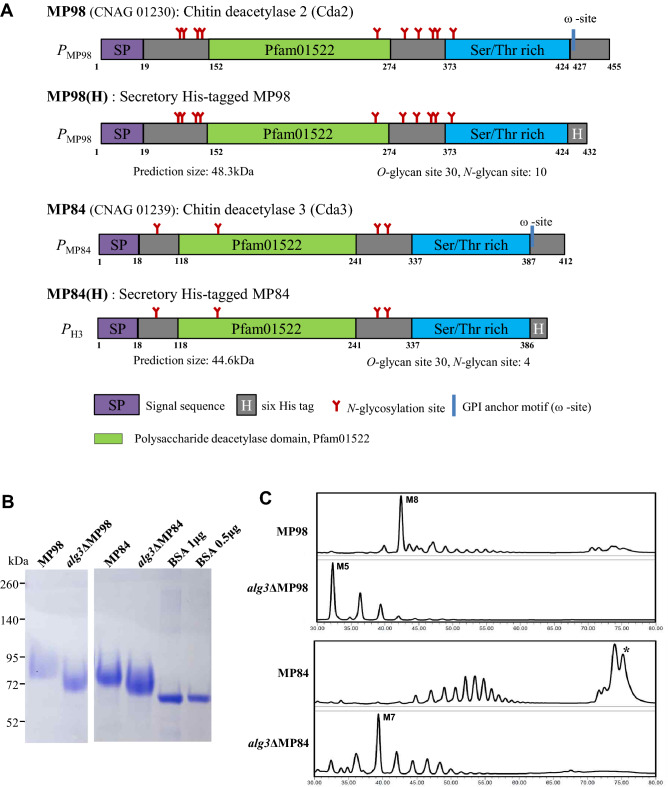
Secretory expression of MP98 and MP84 in *C. neoformans* WT and *alg3*Δ mutant strains. (**A**) Scheme of His-tagged MP98 and MP84 without GPI anchorage. Details about the ORF, promoter, and terminator sequences of the genes encoding MP98 and MP84 were obtained from Broad Institute (https://www.broadinstitute.org). Pfam and SMART were used for domain prediction. Signal sequence and GPI anchor sites were predicted using SignalP 4.1 Server and PredGPI. *N-* and *O-*linked glycosylation sites were predicted on the basis of NetNGlyc 1.0 Server, NetOGlyc 4.0 Server, and YinOYang 1.2 tool analysis. The native MP84 promoter was exchanged with the histone 3 (H3) promoter (*P*_H3_) to direct a high MP84 expression. (**B**) Coomassie Blue staining of the purified MPs from *C. neoformans* WT and *alg3*Δ strains. MPs were purified from cell culture supernatants and analyzed through SDS-PAGE with bovine serum albumin (1 μg BSA) for comparison. The raw data of Coomassie Blue staining in (**B**) was presented in Supplementary Information as Fig. S5. (**C**) HPLC profiles of neutral and acidic (*) *N*-glycans assembled on MP98 and MP84. M8, M5, and M7 represent Man_8_GlcNAc_2_, Man_5_GlcNAc_2_, and Man_7_GlcNAc_2_, respectively.

Cryptococcal MP98 and MP84 contain 455 and 412 amino acids (Supplementary Fig. 1A) and have a chitin deacetylase (CDA) catalytic domain at 152nd–274th and 118th–241st amino acids, respectively (Fig. 1A). Both MP98 and MP84 have a cleavable N-terminal signal sequence and the C-terminal omega site, where glycosylphosphatidylinositol (GPI) is anchored to the protein. MP98 is predicted to have 10 *N*- and 30 *O*-glycosylation sites, respectively, while MP84 has 4 *N*- and 30 *O*-glycosylation sites, respectively. *O*-glycosylation sites are mostly located in the serine/threonine-rich region (Fig. 1A). MP98 and MP84, encoded by the *CDA2* and *CDA3* genes*,* respectively, possess the highly conserved polysaccharide deacetylase domain Pfam01522^31^ and function as a chitin deacetylase that converts chitin into chitosan, which is essential for the cell wall integrity of *C. neoformans*^32^.

As GPI-anchored proteins, MP98 (Cda2) and MP84 (Cda3) are predicted to transverse the plasma membrane and/or attach to the cell wall to deacetylate chitin in the cell wall^33^. In the present study, the GPI anchor motif was exchanged with a polyhistidine tag to achieve the secretory expression of MP98 and MP84 as extracellular proteins. The expression of His-tagged and GPI-anchorless MP98 and MP84 was initially directed under their native promoters. However, the MP84 expression level in recombinant *C. neoformans* cultivated in a YPD medium was too low for the subsequent purification procedure mainly because of the extremely low transcription activity of its native promoter (Supplementary Fig. 1). The MP84 expression level is also 15% less than that of MP98 in *C. neoformans* cells isolated directly from infected human patients^34^. Thus, MP84 was expressed under the control of histone H3 promoter instead of its native promoter (Fig. 1A). The culture medium of *C. neoformans* is highly viscous because of the presence of shed capsule components, such as glucuronoxylomannan, which interferes with the concentration process via centrifugal filters. In this study, MP proteins were expressed in the *C. neoformans cap59*Δ mutant background, where *CAP59*, which is involved in the extracellular trafficking of capsular glucuronoxylomannan^35^, was disrupted to facilitate the purification of MP proteins from the culture supernatant.

The MP proteins were expressed in the wild-type (WT) strain and the *alg3∆* mutant strain, which is defective in core *N*-glycan biosynthesis^20^ and purified from the cell culture supernatant. In SDS-PAGE analysis, the protein bands of MP98 and MP84 secreted from the *alg3∆* cells (*alg3∆*MP98 and *alg3∆*MP84) migrated substantially faster than those from the WT cells; this observation indicated a decrease in size due to the attachment of truncated *N*-glycans (Fig. 1B). As expected, *N*-glycan profiling based on high-performance liquid chromatography (HPLC) confirmed that the overall lengths of *N*-glycans assembled on *alg3∆*MP98 and *alg3∆*MP84 decreased compared with those from WT cells (Fig. 1C). In MP98, the M8 (Man_8_GlcNAc_2_) peak was detected as the major *N*-glycan species in the WT cells. Conversely, the *N*-glycans assembled on MP84 were heavily hypermannosylated, indicating different extents of mannosylation depending on proteins. In the WT cells, substantial portions of *N*-glycans attached to MP84 were detected as acidic *N*-glycans containing xylose phosphate residues^20^. In the *alg3*Δ strain, MP98 and MP84 had truncated core *N*-glycans, mostly M5 and M7 forms as the major species, respectively. Moreover, the acidic *N*-glycans detected in MP84 secreted from the WT strain (indicated by * in Fig. 1C) were not detected in *alg3*ΔMP84. Our previous glycomic analysis of whole cell wall proteins indicated that the truncated core *N*-glycans generated from the *alg3*Δ strain do not possess xylosylphosphotransferase sites^20^; therefore, these observations might explain the disappearance of acid glycans in *alg3*ΔMP84.

### Structural characterization of *N*-linked glycans attached to MP98 and MP84

**Figure 2:**
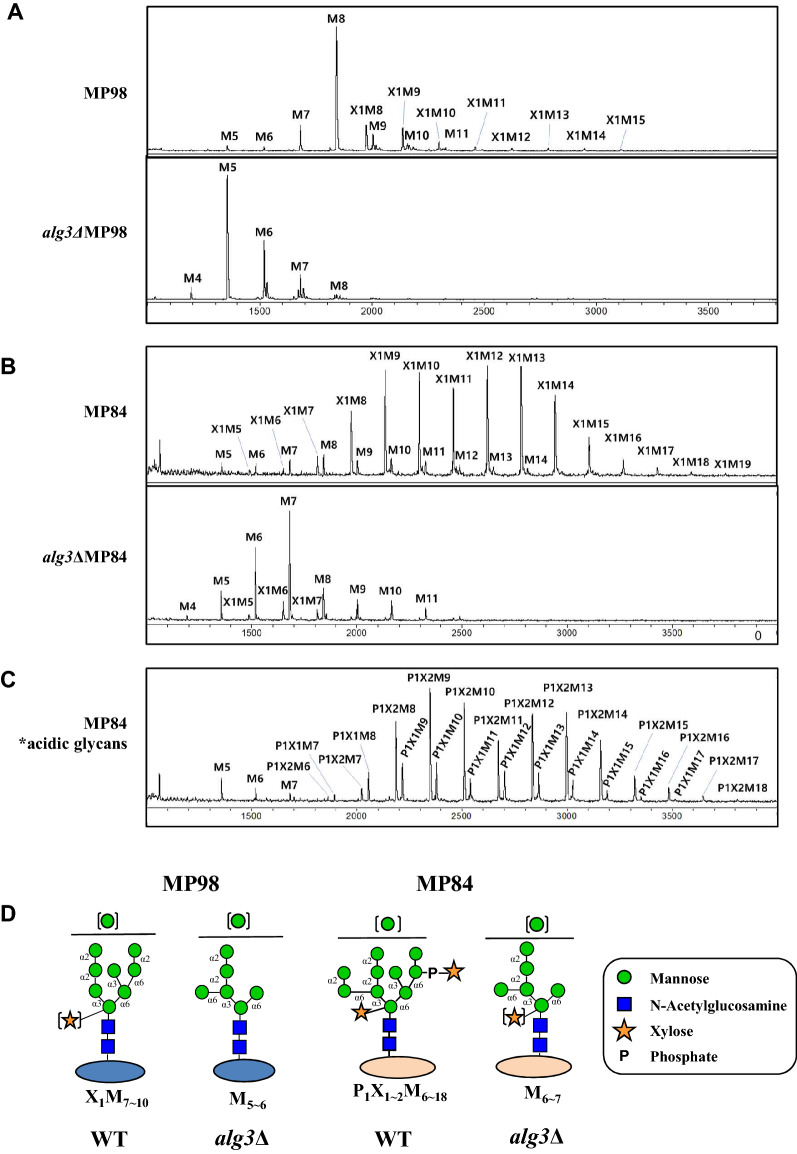
MALDI-TOF profiles of *N*-glycans assembled on MP98 and MP84. (**A**) Analysis of neutral *N*-glycans assembled on MP98 expressed in *C. neoformans* WT and *alg3*Δ mutant strains. (**B**) Neutral *N*-glycans assembled on MP84 expressed in WT and *alg3*Δ strains. (**C**) Acidic *N*-glycans assembled on MP84 expressed in *C. neoformans* WT. (**D**) Structures of *N*-glycans assembled on MP98 and MP84 secreted from WT and *alg3*Δ. P_n_X_n_M_n_ represents Pho_n_Xyl_n_Man_n_GlcNAc_2_ glycans (n = number of phosphates, xyloses, and mannoses).

To obtain more detailed information on the structure of *N*-glycans attached to MP98 and MP84, we performed matrix-assisted laser desorption ionization time-of-flight (MALDI-TOF) analysis of neutral and acidic *N*-glycans separately collected from HPLC fractionation (Fig. 2). As predicted from the HPLC-based glycan profiles, the MALDI-TOF profiles confirmed that the major *N*-glycan species of MP98 is M8, which is composed of eight mannose residues without xylose (Man_8_GlcNAc_2_) in the WT strain; in the *alg3*Δ strain, the major glycan is M5 (Man_5_GlcNAc_2_) carrying five mannose residues (Fig. 2A). In contrast to the low population of *N*-glycans with xylose addition in MP98, the neutral *N*-glycans of MP84 were mostly hypermannosylated with xylose addition (Xyl_1–2_Man_9–18_GlcNAc_2_) in the WT strain as revealed by MALDI-TOF analysis. In the *alg3*Δ strain, the major glycan of MP84 became M7 (Man_7_GlcNAc_2_), along with M4 to M11 forms as minor species that mostly did not have xylose residues (Fig. 2B). The MALDI-TOF profiles of acidic *N*-glycans attached to MP84 confirmed that the acidic glycans (Pho_1_Xyl_1–2_Man_9–18_GlcNAc_2_) were generated by further adding a xylose phosphate residue (Fig. 2C).

α-1,2-Mannosidase and α-1,6-mannosidase treatments sequentially converted most *N*-glycans of MP98 to M5 in the WT strain and to M3 in the *alg3*Δ strain, respectively (Supplementary Fig. 2A). However, the sequential treatment of α-1,2- and α-1,6-mannosidases shifted most *N*-glycans of MP84 to X1M5 (Xyl_1_Man_5_GlcNAc_2_) and X1M8 (Xyl_1_Man_8_GlcNAc_2_) peaks in WT, respectively (Supplementary Fig. 2C). In our previous study on the structural analysis of *N*-glycans assembled on total cell wall MPs from *C. neoformans* WT cells of the serotype A H99 strain, the X1M8 glycan was speculated as an incompletely digested product that was derived from a fraction of glycans lacking α-1,6-mannose extension based on its conversion to X1M5 by additional prolonged digestion with α-1,2-mannosidase^36^. This indicates that the presence of xylose may inhibit the efficient mannose trimming by α-1,2- mannosidases in some glycan structures. In *alg3*Δ, due to the dramatic decrease of xylose addition, most *N*-glycans of MP84 were converted to M3 after the sequential treatment of α-1,2- and α-1,6-mannosidases (Supplementary Fig. 2B,C). Therefore, the results revealed that the *N*-glycans of MP98 and MP84 exhibited different extents of mannosylation with differential addition of xylose and xylose phosphate in the WT cells, indicating protein-specific glycan structures (Fig. 2D). In the *alg3*Δ strain, generating truncated core *N*-glycans, the structure of *N*-glycans was no longer protein specific because of the extremely low efficiency of xylose addition and the lack of mannose residues for xylose phosphate attachment.

### Effect of *N-*glycan truncation on the adhesion of MPs to host cells and uptake by dendritic cells

**Figure 3:**
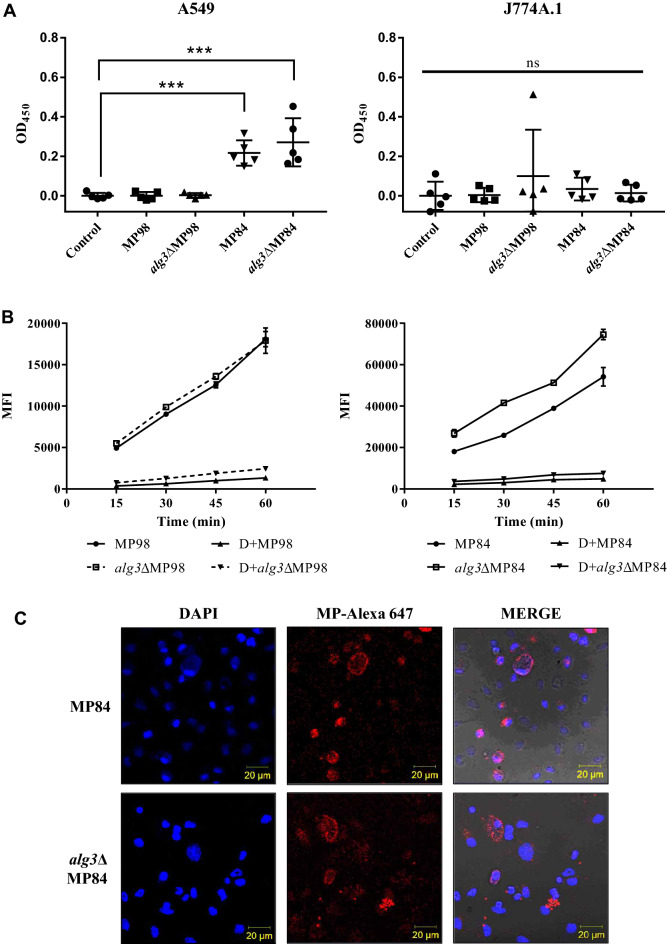
Analysis of cellular adhesion and uptake of MP98 and MP84. (**A**) Adhesion to lung epithelial A594 cells and J774A.1 macrophages. MPs were incubated with mammalian cells for 80 min and analyzed through ELISA. Control is only host cells without incubation with MPs. Significant differences were obtained via one-way ANOVA (*** *P* ≤ 0.001, ns *P* > 0.05). (**B**) Kinetics of the uptake of mannoproteins by BMDCs. MPs (20 μg/ml) labeled with Alexa fluor 647 were incubated with differentiated BMDCs for 15-, 30-, 45-, and 60-min. PBS was used as a negative control. Methyl-α-d-mannopyranoside (+D) was used as a competitive inhibitor of the mannose receptor. The uptake of labeled MPs was analyzed using an Attune NxT flow cytometer with acoustic-assisted hydrodynamic focusing (ThermoFisher) and expressed as the fluorescence geometric mean from an electronically gated live cell population. MFI, mean fluorescence intensity. (**C**) Confocal microscopy analysis of MP uptake by BMDCs. MPs labeled with Alexa fluor 647 dye were incubated with BMDCs for 24 h. The nucleus of BMDCs was stained with 1X DAPI for 1 min and verified through laser scanning confocal microscopy (Carl Zeiss).

In the initial phase of respiratory infection, *C. neoformans* cells are exposed to macrophages and lung epithelial cells. To examine the effect of *N*-glycan truncation on adhesion to host cells, we performed an in vitro adhesion assay by incubating purified MP98 and MP84 with J774A.1 macrophage-like cells and A549 lung epithelial cells for 80 mins (Fig. 3A). Consistent with a previous report on MP84 as an adhesion molecule of *C. neoformans* to A549 lung epithelial cells^4^, our study showed that MP84 had a considerably much higher attachment to lung epithelial cells than MP98. The adhesion of MP84 to lung epithelial cells was evidently much higher than that to macrophages, indicating the cell-specific adherence of MP84 to some extent. However, host cell adherence did not significantly differ between MP84 carrying the WT *N*-glycans and the truncated core *N*-glycans (Fig. 3A).

We further analyzed the uptake of MP98 and MP84 by bone marrow-derived dendritic cells (BMDCs; Fig. 3B) by incubating Alexa fluor 647-labeled MPs with BMDCs. From an early incubation up to 1 h, the uptake of both MPs by BMDCs increased with similar kinetics regardless of the alteration of *N*-glycan structures. *alg3∆*MP98 and *alg3∆*MP84, carrying the truncated core *N*-glycans, were captured by BMDCs as efficiently as those carrying the WT *N*-glycans. This result indicated no apparent effect of the trimmed *N*-glycan structure of MP on adhesion. The efficient internalization of Alexa fluor 647-labeled MPs into BMDCs was verified through confocal microscopy, which showed that the labeled MPs localized mostly to a perinuclear compartment in BMDCs at 15 min co-incubation (Fig. 3C). The treatment with methyl-α-D-mannopyranoside, one of the competitive inhibitors of mannose receptors, effectively inhibited the uptake of MPs. This result demonstrated that recognition by mannose receptors on the surface of BMDCs was involved in the uptake of MP98 and MP84. In addition to the shortened length of *N*-glycans, the lack of xylose and xylose phosphate residues was caused by the truncated core-*N* glycans generated by *ALG3* deletion. Thus, short *N*-glycans containing up to five mannose resides might be sufficient for the efficient binding of MP98 and MP84 to mannose receptors and internalization. Considering a previous report on the inhibitory effect of core β-1,2-xylosylation on glycoprotein recognition by murine C-type lectin receptors^37^, we speculated that the absence of xylose and xylose phosphate residues in *alg3∆*MP98 and *alg3∆*MP84 might compensate for the compromised binding activity of truncated *N*-glycans to the mannose receptor of BMDCs.

### Effect of *N*-glycan truncation on the immune response of BMDCs

**Figure 4:**
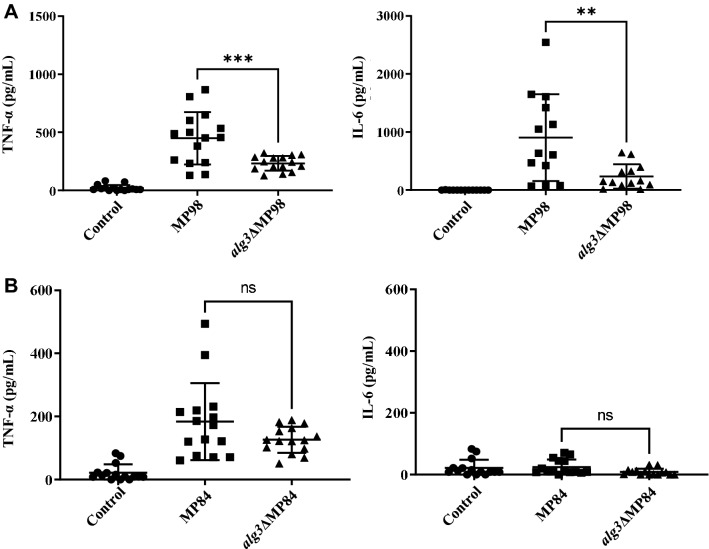
Analysis of the induced cytokine secretion from BMDCs by interaction with MP98 and MP84. After the co-incubation of BMDCs with purified MPs for 24 h, the secreted cytokines in the culture supernatants of BMDCs were examined with a bead-based immunoassay. (**A**) Secreted cytokines after co-incubation with MP98 and *alg3*ΔMP98. (**B**) Secreted cytokines from BMDCs after co-incubation with MP84 and *alg3*ΔMP84. Control is only host cells without incubation with MPs. Significant differences were determined through one-way ANOVA (**P ≤ 0.01; ***P ≤ 0.001, ns *P* > 0.05).

The purified MPs were co-incubated with BMDCs for 24 h to investigate the effect of altered *N*-glycan structure on the host immune response of murine BMDCs, and the cytokines secreted by BMDCs in culture supernatants were analyzed. The secretion of pro-inflammatory cytokines, such as TNF-α and IL-6, noticeably increased in the culture supernatant of BMDCs co-incubated with MP98 carrying the WT *N*-glycans. However, *alg3∆*MP98 showed a significantly decreased activity in inducing cytokine secretion (Fig. 4A). In contrast to co-incubation with MP98, co-incubation with MP84 induced only TNF-α without the detectable induction of IL-6. Moreover, the secretion level of TNF-α by MP84-co-incubated BMDCs was much lower than that by MP98-co-incubated BMDCs (Fig. 4B), implying that MP98 displays higher immune stimulation activity. Although the difference of immune stimulation activity between MP84 and *alg3*ΔMP84 was not as significant as that between MP98 and *alg3*ΔMP98, the induced level of TNF-α was also decreased in the BMDCs co-incubated with *alg3*ΔMP84 (Fig. 4B). Therefore, *N*-glycan structures might play a role in inducing cytokine secretion from BMDCs through interactions with MP98 and MP84.

### Effect of the absence of *N*-glycans on the interaction of MP84 with host cells

**Figure 5:**
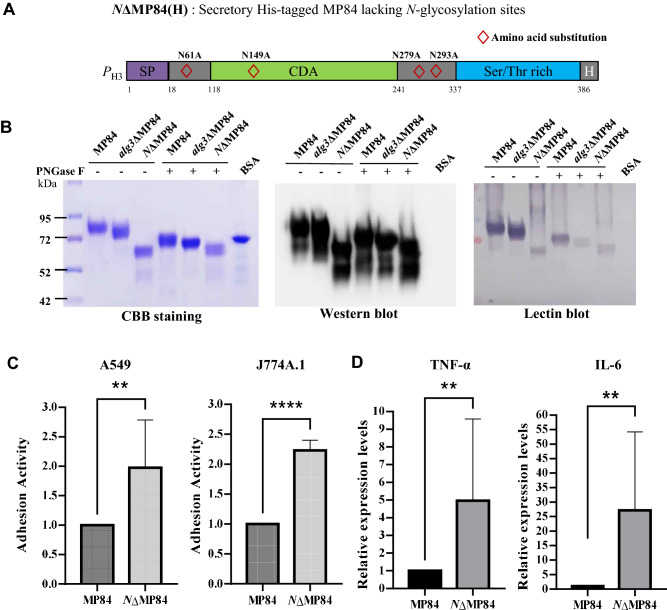
Effect of the absence of *N*-glycans on the interaction of MP84 with host cells. (**A**) Scheme of the engineered MP84 protein lacking all four predicted *N*-glycosylation sites (*N*ΔMP84). All asparagine (Asn) residues in *N*-glycosylation sites were exchanged with alanine (Ala) residues. (**B**) Expression analysis of MP84 lacking *N*-glycosylation sites (*N*ΔMP84). The MP84 secreted by the WT and *alg3*Δ strains, and the MP84 lacking *N-*glycosylation sites were separated via SDS-PAGE and subjected to Coomassie staining, Western blot analysis using anti-His antibody, and lectin-blotting (GNA-AP). The raw data of SDS-PAGE, western blot analysis, and lectin-blotting in (**B**) were presented in Supplementary Information as Fig. S6. (C) Comparison of adhesion activities in host cells between MP84 and *N*ΔMP84. Significant differences were obtained via one-way ANOVA (**P ≤ 0.01, ****P ≤ 0.0001). (**D**) Comparison of the secreted cytokines between BMDCs co-incubated with MP84 and *N*ΔMP84. Significant differences were obtained via one-way ANOVA (**P ≤ 0.01).

The DNA construct encoding the MP84 lacking all *N-*glycosylation sites but retaining most *O*-glycosylation sites (*N*ΔMP84) was generated via protein engineering combined with gene synthesis to maximize the alteration of the *N*-glycan structure (Fig. 5A). *N*-glycosylation sites (N-X-S/T motif) of MP84 were predicted to be located on the 61st, 149th, 279th, and 293rd amino acid residues. Asn codons (AAC or AAT) were changed to Ala codons (GCC or GCT) via fusion PCR and gene synthesis to remove the *N*-glycosylation sites of MP84 (Supplementary Fig. 3A,B). The *N*ΔMP84 construct, containing a C-terminal polyhistidine tag, was expressed under the control of the H3 promoter in the acapsular strain of *C. neoformans*. SDS-PAGE analysis revealed that the *N*ΔMP84 protein showed a dramatically increased mobility, indicating reduced molecular mass compared with that of MP84 expressed in the WT and *alg3*Δ strains. The decreased molecular mass was due to the complete absence of *N*-glycans attached to MP84, which was confirmed by PNGase F treatment (Fig. 5B). Conversely, the size of MP84 secreted in WT and *alg3*Δ strains decreased after PNGase F treatment, but the size of *N*ΔMP84 did not change, demonstrating that all *N*-linked glycosylation sites on MP84 were successfully removed. It is notable that after PNGase F treatment, *alg3*ΔMP84 migrated slightly faster than MP84. Similarly increased electrophoretic migration of MP98 was also observed in our previous study on *C. neoformans alg3* mutation^20^, which might be derived from a reduced extent of occupancy at *N*-glycosylation sites in the *alg3* mutant strains, due to the inefficient transfer of truncated *N*-glycans by oligosaccharyltransferases^38^. After cleavage by PNGase F, the Asn resides at which *N*-glycans attached were converted to aspartic acid (Asp) residues. Considering a little bit larger molecular weight (MW) of Asp compared to Asn, lower *N*-glycan site occupancy in the *alg3∆* mutant strain could generate a decrease in MWs of MP84 and MP98, resulting in faster migration in electrophoresis. Western blot analysis of the culture supernatants (extracellular fraction) and soluble cell lysates (intracellular fraction) of the *C. neoformans* cells expressing His-tagged MP84 indicated the lack of *N*-glycosylation did not affect the secretion efficiency of MP84 (Supplementary Fig. 3C,D). Although the predicted MW of MP84 was 44.6 based on its amino acid sequences, the apparent MW of *N*ΔMP84 on the gel appeared to be larger than the expected size. This finding indicated the presence of post-translational modifications other than *N*-glycosylation, such as *O*-mannosylation, which was confirmed by lectin blotting involving *Galanthus nivalis* lectin (GNA)-alkaline phosphatase (AP) that recognizes mannose residues. Even after the PNGase F treatment, the protein bands of MP84 were detected in all MP84, indicating that they were heavily *O*-mannosylated.

We analyzed the in vitro adhesion of *N*ΔMP84 to macrophages and lung epithelial cells. We unexpectedly found that the abolishment of *N*-glycosylation rather increased the adhesion ability of MP84 protein not only to lung epithelial cells, to which MP84 has preferential adherence, but also to macrophages, to which MP84 has relatively lower adherence (Fig. 5C). Moreover, *N*ΔMP84 induced a significantly higher secretion of pro-inflammatory cytokines from BMDCs than MP84 carrying WT *N*-glycans (Fig. 5D). Particularly, *N*ΔMP84 induced remarkably high levels of IL-6 secretion, which was hardly detected in BMDCs co-incubated with MP84 carrying WT *N*-glycans. Considering that the enforced *N*-glycosylation is generally considered an approach to enhance the immunogenicity of non-glycosylated protein antigens, we suggest that the effect of the abolishment of *N*-glycans on the interaction of MP84 with host cells is complex because of the combined effects exerted by several other factors.

## Discussion

Glycans assembled on the cell surface glycoproteins of fungal pathogens modulate the efficiency of pathogen adhesion to and interaction with host cells during infection^39,40^. The cell wall proteins of various infectious fungi are mostly hypermannosylated and expected to function as immunoreactive molecules to induce host immune responses^21^. The *N*-glycosylation pathway of *C. neoformans* is conserved evolutionarily, but several unique features of *C. neoformans N*-glycans have been described in the structure and biosynthesis pathway^36^. *C. neoformans* has serotype-specific high-mannose-type *N*-glycans with or without a β-1,2-xylose residue, which is attached to the trimannosyl core of *N*-glycans. Moreover, the acidic *N*-glycans of *C. neoformans* contain xylose phosphates attached to mannose residues in the *N*-glycan core and outer mannose chains. Although the outer chains of *N*-glycans are dispensable for the virulence of *C. neoformans*, the intact core *N*-glycan structure is required for *C. neoformans* pathogenicity in the systematic analysis of *alg3∆*, *alg9∆*, and *alg12∆* mutant strains that have defects in lipid-linked *N-*glycan assembly^20^. In the present study, we investigated the effect of the altered structure of *N*-glycans assembled on cryptococcal cell surface MP98 and MP84, known as the T-cell antigen and the adhesion molecule in host lung epithelial cells, respectively^4,26^, by analyzing the interaction of purified fungal MPs with host cells.

Intriguingly, our data on the *N*-glycan structure analysis of MP98 and MP84 revealed significant differences in their *N*-glycan patterns, indicating the protein-specific *N*-glycan structures with different extents of mannosylation and addition of xylose and xylose phosphate residues (Figs. 1 and 2). In the WT strain, although MP98 had *N*-glycans carrying eight mannoses (M8) as major species, MP84 had long hypermannosylated glycans decorated not only with xylose but also with xylose phosphate residues, generating acidic glycans. As functional chitin deacetylases, MP98 (Cda2) and MP84 (Cda3) show very similar structural organization by possessing a cleavable *N*-terminal signal sequence, a C-terminal omega site for GPI anchoring, and the highly conserved polysaccharide deacetylase domain Pfam01522. Although MP98 and MP84 had similar predicted MWs, they showed only 30% identity in the overall amino acid sequences (Supplementary Fig. 1A) and notable differences in *N*-glycosylation sites. MP98 had 10 predicted *N*-glycosylation sites, whereas MP84 had only four predicted *N-*glycosylation sites. Considering that the further modification of core-*N*-glycans, such as addition of extra mannose, xylose and xylose phosphate resides, was mediated by Golgi-resident enzymes, we speculated that the different three-dimensional structures and trafficking rates of the two MPs during secretion might be attributed to differential modification by processing enzymes in the Golgi; consequently, protein-specific *N*-glycan structures were formed.

The recombinant *alg3*ΔMP98 and *alg3*ΔMP84, carrying truncated core *N*-glycans, had M5 and M7 lacking xylose and xylose phosphate residues as the major *N*-glycan species, respectively (Fig. 2). In comparison with the MPs carrying the WT *N*-glycans, *alg3*ΔMP98 and *alg3*ΔMP84 retained almost equivocal adhesion affinities to host cells, indicating that the altered core *N*-glycan structure did not affect the adhesion ability of MPs (Fig. 3A). This result was consistent with our previous observations that the adhesion efficiency in lung epithelial cells has no significant differences between WT cells and *alg3*Δ mutant cells^20^. *alg3*ΔMP98 and *alg3*ΔMP84 also showed comparable uptake kinetics by BMDCs, which rapidly captured the fluorescent-labeled MPs (Fig. 3B,C). This result implied that the truncated core *N*-glycans containing more than five mannose residues are still efficiently recognized by the mannose receptor of dendritic cells^41,42^. Considering that MP98 and MP84 have 30 *O-*mannosylation sites (Fig. 1), we could speculate that the presence of short-chain *O*-linked terminal mannose residues at the serine/threonine-rich C-terminus could contribute to the retainment of the binding capacity to mannose receptors. A previous study reported that multiple lectin receptors on dendritic cells recognize *C. neoformans* MPs, which may potentially contribute to glycoprotein-specific T cell immunity^43^. However, *alg3*ΔMP98 showed significantly decreased activities in inducing immune responses from BMDCs, although the decrease was less evident in *alg3*ΔMP84 with low immune stimulation activity (Fig. 4), consistent with our previous report on the decreased TNF-α level in the BMDCs infected with the *alg3*Δ strain^20^. This finding confirmed that truncated core *N*-glycans were less efficient in activating host immune responses than *N*-glycans carrying a WT structure.

We further investigated the effect of the absence of *N*-glycans by generating the *N*ΔMP84 protein through the removal of all four predicted *N*-glycosylation sites. In the present study, we replaced Asn codons in the predicted sites with Ala codons to minimize the effect of amino acid substitution on the structure of MP84. Glutamine (Glu) shares more similar chemical properties to Asn in the aspect of bearing an amide side chain. However, reflecting its bigger size than Asn due to the presence of an extra methyl group, the in silico 3D structure of MP84 with Glu substitution was predicted to be bulkier with a disorganized C-terminus compared to those of the original MP84 protein and Ala-substituted MP84 (Supplementary Fig. 3A). Previous works reported that there were no functional differences in endothelial lipases in which the *N*-glycosylation sites were mutated to either Ala or Gln^44^. In addition, a work by Valliere-Douglass et al. identified the presence of a glutamine-linked protein glycosylation motif in human recombinant IgG proteins^45^. Considering that Ala substitution caused the less change of 3D-structure (Supplementary Fig. 3A) and could avoid possible ectopic glycosylation at Gln, Ala substitution was chosen to generate the *N*ΔMP84 protein. In contrast to the effect of the truncated core *N*-glycans, the complete abolishment of *N*-glycans exerted quite unexpected effects; thus, the adhesion affinity to host cells dramatically increased nonspecifically, and the induced activity of cytokine secretion from BMDCs was higher than that of MP84 carrying WT *N*-glycans (Fig. 5). These results strongly indicated that MP84 protein itself had a high adhesion capacity and a strong immune-stimulating activity. The recombinant proteins of *C. neoformans* Cda2 (MP98) and Cda3 (MP84) expressed in *Escherichia coli* was shown to induce protective immunity in mice against *C. neoformans* infection, indicating that the recombinant aglycosylated MP98 and MP84 can inherently modulate host immune responses^46^. Another recent study on the efficient protection of mice against experimental cryptococcosis by chemically synthesized peptides^47^ strongly indicated that the protein epitopes of MPs are intrinsically immunodominant. Most cryptococcal MPs have multiple potential sites for *O*-linked glycans, and the removal of *O*-linked carbohydrates by $\upbeta$-elimination dramatically decreases T-cell responses compared with those that do not have apparent alteration of immunoreactivity after PNGase F treatment^48^. Thus, the absence of *N*-glycans might increase the exposure of more immunogenic *O*-glycans attached to MP84. Altogether, our data strongly suggest that the *N*-glycans of MP84 might have a modulatory role in decreasing nonspecific interactions with host cells and avoiding the hyperactivation of host immune responses by covering immunogenic components on the fungal cell surface.

After the cell adhesion activity unexpectedly increased upon abolishing *N*-glycans in MP84 (*N*ΔMP84), MP98 lacking *N*-glycans was prepared via PNGase F treatment under non-denatured conditions (Supplementary Fig. 4). The PNGase F-treated MP98 was purified and analyzed for its adhesion activity by co-incubating with host cells. Similar to the observations with *N*ΔMP84, the *N*-glycan-removed MP98 showed approximately tenfold increased adhesion to A549 cells and about 60-fold increased adhesion to J774A.1 macrophage cells compared with those in MP98 with WT *N*-glycans (Supplementary Fig. 4). In contrast to MP98 carrying WT *N*-glycans, which showed low adhesion activity to host cells, the *N*-glycan-removed MP98 became highly adhesive to lung epithelial cells and macrophage cells without cell-type specificity. This indicated that the presence of *N*-glycans prevented MP98 from attaching nonspecifically to host cells. A previous study on two plasma proteins, α1-acid glycoprotein (AGP) and haptoglobin (Hp), also revealed such varying effects of altered *N*-glycan structures on the interaction of plasma proteins^49^. The increased antennae branching and terminal fucosylation of *N*-glycans reduce the drug-binding affinity of AGP, but opposite effects of fucosylation and *N*-glycan branching occur in Hp–Hp interactions^49^. Several experimental data have also demonstrated that glycosylation can interfere with antigen processing and presentation^50^.

In conclusion, our data revealed the structure-dependent effects of *N*-glycans on the function of cryptococcal MP, and the altered structures of *N*-glycans attached to MP98 and MP84 exerted complicated effects on host cell interactions. Specifically, truncated core *N*-glycans, which are composed of five or seven mannose residues without xylose and xylose phosphate residues, did not affect the adherence of MPs to host cells, but resulting in a decreased immune-stimulating activity. The reduced host immune response in the interaction with *alg3*ΔMP98 and *alg3*ΔMP84 was consistent with the general notion that hypermannosylated fungal *N*-glycan structures are highly immunogenic to induce immune responses of host cells. However, the complete abolishment of *N*-glycans from MP84 and MP98 remarkably enhanced their activities with host cells; this result indicated that the increased immune-stimulating activity of the complete absence of *N*-glycans might be attributed to the increased exposure of protein epitopes or immunoreactive *O*-glycans of MPs to host cells. Therefore, recombinant MPs without *N*-glycans but still having heavily mannosylated *O*-glycans could be a better vaccine candidate than endogenous MP98 and MP84 carrying WT *N*-glycans. However, further studies should be performed to examine the defined roles of *O*-glycan structures of MPs in the interaction with host cells and provide insights into the development of new vaccines for cryptococcosis infection by modulating the structures of glycans assembled on MPs.

## Materials and methods

### Yeast strains, culture conditions, plasmids, and primers

Yeast cells were generally cultured in YPD medium (1% yeast extract, 2% bacto peptone, and 2% glucose) at 28 °C. For cell wall stability before the biolistic transformation of *C. neoformans*, YPD agar medium (1% yeast extract, 2% bacto peptone, 2% glucose, and 2% agar) containing 1 M sorbitol was used. YPD agar medium containing 200 μg/ml G418 (Duchefa, Netherlands) was used for *C. neoformans* transformant selection. *E. coli* TOP10 (Invitrogen, USA) was used to clone recombinant DNA and cultured in LB medium (0.5% yeast extract, 1% bacto tryptone, and 1% NaCl) containing 100 μg/ml ampicillin (Duchefa, Netherlands). Yeast strains and plasmids are listed in Table 1. The primers used in this study are listed in Supplementary Table 1.

**Table 1:** List of *C. neoformans* strains and plasmids used in this study.

Strains and plasmids	Description	Reference
**Strains**		
*Cryptococcus neoformans*		
H99	*MATα* (Serotype A)	^51^
*cap59*Δ	*MATα* Cn00721::*HYB*	^20^
*alg3*Δ *cap59*Δ	*MATα* Cn05412::*NAT#159* Cn00721::*HYB*	^20^
MP98(H)/*cap59*Δ	*MATα* Cn00721::*HYB* Cn01230(H)::*NEO*	This study
MP98(H)/*alg3*Δ *cap59*Δ	*MATα* Cn05412::*NAT#159* Cn00721::*HYB* Cn01230(H)::*NEO*	This study
MP84(H)/*cap59*Δ	*MATα* Cn00721::*HYB P*_*H3*_*::*Cn01239::*NEO*	This study
MP84(H)/*alg3*Δ *cap59*Δ	*MATα* Cn05412::*NAT#159* Cn00721::*HYB P*_*H3*_*::*Cn01239::*NEO*	This study
*N*ΔMP84(H)/*cap59*Δ	*MATα* Cn05412::*NAT#159* Cn00721::*HYB P*_*H3*_*::*Cn01239N(61, 149, 279, 293)A::*NEO*	This study
**Plasmids**		
pJAFS1	Modified pJAF12 containing *NEO*^r^ as a selection marker	^36^
pJAFS1-CNAG01230His	pJAFS1-based expression vector for six histidine-tagged MP98	^20^
pJAFS1-pH3CNAG01239His	pJAFS1-based expression vector for six histidine-tagged MP84 under the H3 promoter	This study
pJAFS1pH3CNAG01239(N del)His	pJAFS1-based expression vector for six histidine-tagged MP84 without *N*-glycans under the H3 promoter	This study

### Construction of recombinant *C. neoformans* secreting His-tagged MP84

The PCR fragment of CNAG 01239 Terminator (489 bp) was obtained from the H99 genomic DNA by using the primers Cn_01239_T_F_EcoRV and Cn_01239_T_B_NotI (Supplementary Table 1) and subcloned into EcoRV/NotI-digested pJAFS1 (Table 1) to generate pJAFS1_CNAG_01239Ter and thus construct the expression vector of 6His-tagged MP84. The DNA fragments containing the *N*-truncated MP84 ORFs with the 6His codon as the C-terminal histidine tag without the GPI anchor (1.3 kb) was PCR amplified with the primers Cn_01239_O_F_Xho and Cn_01239_O_B_EcoRV (Table S1). The PCR product of the truncated MP84 ORF was digested with XhoI/EcoRV and ligated into XhoI/EcoRV-digested pJAFS1_CNAG_01239Ter, generating pJAFS1-CNAG 01230His. Subsequently, the promoter of histone H3 (CNAG 06745), amplified via PCR by using the primer set H3 promoter_F and Infusion MP84_R, was inserted in front of the MP84 ORF in pJAFS1-CNAG 01230His via Infusion (Takara Bio, USA); thus, pJAFS1pH3CNAG012396His was obtained.

For the expression of MP84 without *N*-glycans, the asparagine (Asn, N) codon in four predicted *N-*glycosylation sites were replaced with alanine (Ala, A) codon in MP84 ORF via site-directed mutagenesis and synthetic DNA fragment (Supplementary Fig. 4). The mutated MP84 ORF carrying the N(61, 149, 279, 293)A replacement was expressed under the control of H3 promoter in the pJAFS1 backbone, generating pJAFS1pH3CNAG01239(N del)His. The resultant vectors pJAFS1pH3CNAG 01239His and pJAFS1pH3CNAG01239(N del)His were digested at NsiI and delivered into *cap59*Δ and *alg3*Δ *cap59*Δ cells (Table 1) via a biolistic particle delivery system for integration into the chromosomal H3 promoter region through homologous recombination. Transformants were selected on a YPD plate containing 100 μg/ml neomycin, screened through PCR, and analyzed with western blot by using an anti-His antibody to detect His-tagged MP84.

### Protein purification and western blotting

Recombinant *C. neoformans* cells expressing His-tagged secretory MP98 or MP84 were pre-cultured in 2 ml of YPD at 28 °C overnight. They were inoculated in 200 ml of YPD with an OD_600_ of 0.5 in a shaking incubator (220 rpm) at 28 °C for 24 h. A cell-free supernatant medium was isolated through centrifugation (4000 rpm, 4 °C, 10 min) and then filtered using a 0.22 μm nitrocellulose membrane (Millipore, USA) and a vacuum pump. The supernatant containing the secreted MP was concentrated in Amicon Ultra-15 Centrifugal Filter units (50 kDa, Millipore, USA) through centrifugation (4000 rpm, 4 °C, 10 min). The concentrated solution with His-trap binding buffer (20 mM sodium phosphate, 0.5 M NaCl, 40 mM imidazole in triple distilled water (TDW), pH 7.4, filtered with 0.45 μm syringe filter) was incubated in a rotated column with activated Ni Sepharose beads (GE Healthcare, USA) at 4 °C overnight. Ni beads were washed with 2 ml of His-trap elution buffer (20 mM sodium phosphate, 0.5 M NaCl, 500 mM imidazole in TDW, pH 7.4, filtrated by 0.45 μm syringe filter) four times to elute the His-tagged MPs. The eluted MPs were dialyzed in PBS (137 mM NaCl, 2.7 mM potassium chloride, 10 mM disodium hydrogen phosphate, 1.8 mM potassium dihydrogen phosphate, pH 7.4, autoclaved) at 4 °C for 2 days overnight and concentrated with Amicon Ultra-15 Centrifugal Filter units.

For western blotting, proteins were analyzed on 8% polyacrylamide gel and transferred to polyvinylidene fluoride (PVDF) membranes. Anti-His mouse antibody (1:1000 dilution, Santa Cruz Biotechnology, USA) was treated and incubated at 4 °C overnight. The washed membrane was treated with anti-mouse IgG antibody conjugated with alkaline phosphatase (1:10,000 dilution, Santa Cruz Biotechnology, USA) and incubated at RT for 1 h. Afterward, an AP conjugate substrate kit (Bio-Rad) was used to induce color change.

### HPLC and MALDI-TOF-based *N*-glycan structure analysis

*N*-glycans were analyzed through HPLC and MALDI-TOF^20^. Briefly, *N*-glycans were separated from 15 μg of MP by PNGase F (New England Biolabs, UK). After purification with a Carbograph solid-phase extraction column, the separated *N*-glycans were labeled with anthranilic acid (2-AA; Sigma-Aldrich, USA) and purified. Each dried *N*-glycan sample was dissolved in 50 μl of HPLC water and analyzed using normal-phase HPLC with a Shodex Asahipak NH2P-50 4E column (0.43 × 25 cm, SHOWA DENKO K.K., Japan), in 70% solvent A (1% tetrahydrofuran [THF] and 2% acetic acid in 100% Acetonitrile) and 30% solvent B (1% THF, 5% acetic acid, and 3% triethylamine in HPLC water), for 90 min and detected with a fluorescence detector at 360 and 425 nm wavelengths.

For MALDI-TOF analysis, *N-*glycans fractionated via HPLC were collected and dried. The matrix solution (the same ratio of 6-Aza-2-thiothymine solution and 2,5-Dihydroxy-benzoic acid solution) was mixed with the same volume of *N-*glycan sample. The sample was spotted on an MSP 96 polished-steel target (Bruker, Germany), and the crystallized sample was analyzed via MALDI-TOF (Bruker, Germany) in a linear negative mode.

### Cultivation of A549 and J774A.1 cells

A549 (human lung epithelial cells) and J774A.1 (BALB/C mouse macrophages) were acquired from the Korean Cell Line Bank of Seoul National University. Each cell was cultured in cell culture medium (RPMI 1640 complete medium; HyClone, USA) supplemented with 10% fetal bovine serum (FBS, Access Biologicals, USA) and 1% penicillin/streptomycin (Gibco, USA) in a humidified incubator with 5% CO_2_ conditions at 37 °C. The medium was changed every 1–2 days. A Trypsin–EDTA solution (Welgene, Taiwan) and a scraper were used to scrape A549 and J774A.1 cells, respectively.

### In vitro adhesion assay of MP98 and MP84 in lung epithelial cells

The cultured mammalian cells were seeded in a 96-well plate at a density of 6.0 × 10^4^ A549 cells per well and 1.25 × 10^4^ J774A.1 cells per well in the cell culture medium for 1 day, respectively. After serum starvation, 1 μg of the purified and quantified MPs was added to the cells with FBS-free RPMI and incubated in an incubator containing 5% CO_2_ at 37 °C for 80 min. The cells were washed with PBS-T four times and fixed with PBS-based 4% paraformaldehyde for 30 min. After each well was washed, the cells were blocked with 0.5% casein in PBS-T at 37 °C for 1 h and washed with PBS-T. After being treated with mouse anti-His monoclonal antibody (1:3000 dilution, Santa Cruz Biotechnology, USA) in PBS-T containing 0.5% casein at RT for 1 h, the cells were washed and incubated with HRP-conjugated anti-mouse IgG antibody (1:2000 dilution, Santa Cruz Biotechnology, USA) at RT for 1 h. After each well was washed, 100 μl of TMB-blotting substrate solution (Sigma-Aldrich, USA) was added to the cells, which were then incubated for 15 min to develop a color change. Then, 100 μl of the stop solution (2 M H_2_SO_4_) was added to stop the reaction. The absorbance of each well was measured using a UVM 340 microplate reader at 450 nm wavelength.

### Analysis of in vitro protein uptake by bone marrow dendritic cells

BMDCs were isolated from murine femur and tibiae (C57BL/6 J, 7 W, male)^20^. Progenitor cells were incubated in a culture medium with 20 ng/ml GM-CSF (PeproTech, USA) at 37 °C for 6 days to obtain the differentiated BMDCs. A fresh DC differentiation medium was added 3 days after the bone marrow cells differentiated into dendritic cells and floated. Alexa Fluor 647 protein labeling kit (Invitrogen, USA) was used to prepare the labeled MP proteins in accordance with the manufacturer’s instructions. Briefly, MP proteins were mixed with Alexa fluor 647 dye and incubated while stirring at RT for 1 h. The labeled proteins were purified using a purification column. Afterward, 20 μg/ml labeled MPs in a RPMI medium containing 10% HI-FBS was added to the differentiated BMDCs in a 24-well plate at a density of 6 × 10^5^ cells per well and then incubated in the absence and presence of 50 mM methyl-α-D-mannopyranoside (Sigma-Aldrich, USA) for 60 min. After the uptake at the indicated time points, the cells were washed and analyzed with an Attune NxT flow cytometer with acoustic-assisted hydrodynamic focusing (Thermo Fisher Scientific, USA). Uptake was expressed as the fluorescence geometric mean from an electronically gated live cell population.

### Analysis of cytokine secretion from dendritic cells

The differentiated BMDCs were seeded in a 96-well plate (u-type) at a density of 1 × 10^5^ cells per well in a RPMI medium containing 10% HI-FBS and incubated for 1 h. Each of the purified MP proteins (20 μg/ml) in the RPMI medium containing 10% HI-FBS was added to BMDCs, which were then incubated for 24 h. After the supernatant was extracted, a bead-based immunoassay was performed using a LEGENDplex bead capturing cytokine kit (BioLegend, USA) to measure the cytokine profile in accordance with the manufacturer’s instruction.

## Supplementary Information

Supplementary Information.

## Data Availability

All data generated or analyzed during this study are included in this published article and its supplementary information file.

## References

[1] Singh N (2007). *Cryptococcus neoformans* in organ transplant recipients: Impact of calcineurin-inhibitor agents on mortality. J. Infect. Dis..

[2] Rajasingham R (2017). Global burden of disease of HIV-associated cryptococcal meningitis: An updated analysis. Lancet Infect. Dis..

[3] Velagapudi R, Hsueh Y-P, Geunes-Boyer S, Wright JR, Heitman J (2009). Spores as infectious propagules of *Cryptococcus neoformans*. Infect. Immun..

[4] Teixeira PAC, Penha LL, Mendonça-Previato L, Previato JO (2014). Mannoprotein MP84 mediates the adhesion of *Cryptococcus **neoformans* to epithelial lung cells. Front. Cell. Infect. Microbiol..

[5] Evans RJ (2015). Cryptococcal Phospholipase B1 Is required for intracellular proliferation and control of titan cell morphology during macrophage infection. Infect. Immun..

[6] Ma, H. M., R. C. in *Advances in Applied Microbiology* Vol. 67 Ch. 5 Virulence in *Cryptococcus* Species, 131–190 (Academic Press, 2009). 10.1016/S0065-2164(08)01005-8 19245939

[7] Sabiiti W, May RC (2012). Mechanisms of infection by the human fungal pathogen *Cryptococcus neoformans*. Future Microbiol..

[8] Gaylord EA, Choy HL, Doering TL (2020). Dangerous liaisons: interactions of *Cryptococcus neoformans* with host phagocytes. Pathogens.

[9] García-Rodas R, Zaragoza O (2012). Catch me if you can: Phagocytosis and killing avoidance by *Cryptococcus neoformans*. FEMS Immunol. Med. Microbiol..

[10] Charlier C (2009). Evidence of a role for monocytes in dissemination and brain invasion by *Cryptococcus neoformans*. Infect. Immun..

[11] Santangelo R (2004). Role of extracellular phospholipases and mononuclear phagocytes in dissemination of cryptococcosis in a murine model. Infect. Immun..

[12] Santiago-Tirado, F. H. *et al.* Trojan horse transit contributes to blood–brain barrier crossing of a eukaryotic pathogen. *mBio* **8**, e02183–02116 (2017). 10.1128/mBio.02183-16 PMC5285505 28143979

[13] Oliveira AP, Sauer U (2012). The importance of post-translational modifications in regulating *Saccharomyces cerevisiae* metabolism. FEMS Yeast Res..

[14] Cherepanova N, Shrimal S, Gilmore R (2016). *N*-linked glycosylation and homeostasis of the endoplasmic reticulum. Curr. Opin. Cell Biol..

[15] Ohtsubo K, Marth JD (2006). Glycosylation in cellular mechanisms of health and disease. Cell.

[16] West L (2013). Differential virulence of *Candida glabrata* glycosylation mutants. J. Biol. Chem..

[17] Frieman MB, McCaffery JM, Cormack BP (2002). Modular domain structure in the *Candida glabrata* adhesin Epa1p, a beta1,6 glucan-cross-linked cell wall protein. Mol. Microbiol..

[18] Chong KTK, Woo PCY, Lau SKP, Huang Y, Yuen KY (2004). *AFMP2* encodes a novel immunogenic protein of the antigenic mannoprotein superfamily in *Aspergillus fumigatus*. J. Clin. Microbiol..

[19] Lee DJ (2015). Unraveling the novel structure and biosynthetic pathway of *O*-linked glycans in the golgi apparatus of the human pathogenic yeast *Cryptococcus neoformans*. J. Biol. Chem..

[20] Thak, E. J. *et al.* Core *N*-glycan structures are critical for the pathogenicity of *Cryptococcus neoformans* by modulating host cell death. *mBio* **11**, e00711–00720 (2020). 10.1128/mBio.00711-20 PMC7218283 32398313

[21] Rodrigues M, Nimrichter L (2012). In good company: Association between fungal glycans generates molecular complexes with unique functions. Front. Microbiol..

[22] Chaka W (1997). Induction of TNF-alpha in human peripheral blood mononuclear cells by the mannoprotein of *Cryptococcus neoforman*s involves human mannose binding protein. J. Immunol..

[23] Pietrella D (2001). Role of mannoprotein in induction and regulation of immunity to *Cryptococcus neoformans*. Infect. Immun..

[24] Pietrella D, Corbucci C, Perito S, Bistoni G, Vecchiarelli A (2005). Mannoproteins from *Cryptococcus neoformans* promote dendritic cell maturation and activation. Infect. Immun..

[25] Levitz SM, Nong SH, Mansour MK, Huang C, Specht CA (2001). Molecular characterization of a mannoprotein with homology to chitin deacetylases that stimulates T cell responses to *Cryptococcus neoformans*. Proc. Natl. Acad. Sci..

[26] Biondo C (2005). Characterization of two novel cryptococcal mannoproteins recognized by immune sera. Infect. Immun..

[27] Biondo C (2006). Identification of major proteins secreted by *Cryptococcus neoformans*. FEMS Yeast Res..

[28] Dan JM, Wang JP, Lee CK, Levitz SM (2008). Cooperative stimulation of dendritic cells by *Cryptococcus **neoformans* mannoproteins and CpG oligodeoxynucleotides. PLoS ONE.

[29] Taylor-Smith LM (2017). Cryptococcus–epithelial interactions. J. Fungi.

[30] Rizzo J (2021). *Cryptococcus* extracellular vesicles properties and their use as vaccine platforms. J. Extracell. Vesicles.

[31] Lee S, Kang HA, Eyun SI (2020). Evolutionary analysis and protein family classification of chitin deacetylases in *Cryptococcus neoformans*. J Microbiol..

[32] Baker LG, Specht CA, Donlin MJ, Lodge JK (2007). Chitosan, the deacetylated form of chitin, is necessary for cell wall integrity in *Cryptococcus neoformans*. Eukaryot. Cell.

[33] Gilbert, N. M., Baker, L. G., Specht, C. A., Lodge, J. K. & Pirofski, L.-A. A glycosylphosphatidylinositol anchor is required for membrane localization but dispensable for cell wall association of chitin deacetylase 2 in *Cryptococcus neoformans*. *mBio* **3**, e00007–00012 (2012). 10.1128/mBio.00007-12 PMC3280450 22354955

[34] Chen Y. *et al*. The *Cryptococcus neoformans* transcriptome at the site of human meningitis. *mBio* **5**, e01087–13 (2014). 10.1128/mBio.01087-13 PMC3950508 24496797

[35] García-Rivera J, Chang YC, Kwon-Chung KJ, Casadevall A (2004). *Cryptococcus neoformans CAP59* (or Cap59p) is involved in the extracellular trafficking of capsular glucuronoxylomannan. Eukaryot. Cell.

[36] Park JN (2012). Unraveling unique structure and biosynthesis pathway of *N*-linked glycans in human fungal pathogen *Cryptococcus neoformans* by glycomics analysis. J. Biol. Chem..

[37] Brzezicka K (2016). Influence of core β-1,2-xylosylation on glycoprotein recognition by murine C-type lectin receptors and its impact on dendritic cell targeting. ACS Chem. Biol..

[38] Aebi M (1996). Cloning and characterization of the *ALG3* gene of *Saccharomyces cerevisiae*. Glycobiology.

[39] Hall RA (2013). The Mnn2 mannosyltransferase family modulates mannoprotein fibril length, immune recognition and virulence of *Candida albicans*. PLoS Pathog..

[40] Gómez-Gaviria M, Vargas-Macías AP, García-Carnero LC, Martínez-Duncker I, Mora-Montes HM (2021). Role of protein glycosylation in interactions of medically relevant fungi with the host. J. Fungi.

[41] Pontow SE, Kery V, Stahl PD (1992). Mannose receptor. Int. Rev. Cytol..

[42] Garrett, W. S., & Mellman, I. in *Dendritic Cells* Ch. 16—Studies of Endocytosis, 213-cp211 (Academic Press, 2001).

[43] Mansour MK, Latz E, Levitz SM (2006). *Cryptococcus neoformans* glycoantigens are captured by multiple lectin receptors and presented by dendritic cells. J. Immunol..

[44] Miller GC (2004). Role of *N*-linked glycosylation in the secretion and activity of endothelial lipase. J. Lipid Res..

[45] Valliere-Douglass JF (2010). Glutamine-linked and non-consensus asparagine-linked oligosaccharides present in human recombinant antibodies define novel protein glycosylation motifs. J. Biol. Chem..

[46] Specht, C. A. *et al.* Vaccination with recombinant *Cryptococcus* proteins in glucan particles protects mice against cryptococcosis in a manner dependent upon mouse strain and cryptococcal species. *mBio* **8**, e01872–01817 (2017). 10.1128/mBio.01872-17 PMC5705919 29184017

[47] Specht C. A., *et al*. Protection of mice against experimental cryptococcosis by synthesized peptides delivered in glucan particles. *mBio*. **13**, e0336721 (2022). 10.1128/mbio.03367-21 PMC8725579 35089095

[48] Mansour MK, Schlesinger LS, Levitz SM (2002). Optimal T cell responses to *Cryptococcus neoformans* mannoprotein are dependent on recognition of conjugated carbohydrates by mannose receptors. J. Immunol..

[49] Wu D, Struwe WB, Harvey DJ, Ferguson MAJ, Robinson CV (2018). *N*-glycan microheterogeneity regulates interactions of plasma proteins. Proc. Natl. Acad. Sci..

[50] Zhou JY, Oswald DM, Oliva KD, Kreisman LSC, Cobb BA (2018). The glycoscience of immunity. Trends Immunol..

[51] Perfect JR, Ketabchi N, Cox GM, Ingram CW, Beiser CL (1993). Karyotyping of *Cryptococcus neoformans* as an epidemiological tool. J. Clin. Microbiol..

