# Supplementary material for: Effects of altered N-glycan structures of Cryptococcus neoformans mannoproteins, MP98 (Cda2) and MP84 (Cda3), on interaction with host cells
Source: Sci Rep. 2023 Jan 20;13:1175. doi: 10.1038/s41598-023-27422-9 (PMC9859814; doi:10.1038/s41598-023-27422-9)
Supplement: Supplementary file 1 — Supplementary Information. [file 41598_2023_27422_MOESM1_ESM.docx]

**Supplementary Information**

**Effects of altered *N*-glycan structures of *Cryptococcus neoformans* mannoproteins, MP98 (Cda2) and MP84 (Cda3), on interaction with host cells**

Su-Bin Lee^1, #^, Catia Mota^1, #^, Eun Jung Thak^1^, Jungho Kim^1^, Ye Ji Son^1^, Doo-Byoung Oh^2, 3^, and Hyun Ah Kang^1*^

^1^Department of Life Science, College of Natural Science, Chung-Ang University, Seoul 156-756, South Korea

^2^ Korea Research Institute of Bioscience and Biotechnology (KRIBB), Daejeon 34141, South Korea

^3^Department of Biosystems and Bioengineering, KRIBB School, University of Science and Technology (UST), Daejeon 34113, South Korea

^#^ Contributed equally to this work

*For correspondence. E-mail: hyunkang@cau.ac.kr; Tel.: +82-2-820-5863; Fax: +82-2-825-5206

Contents

Supplementary Table S1

Supplementary Figures S1–S6

Supplementary References

**Supplementary Table 1. List of primers used in this study**

| **Primers** | | **Sequence description** | | **Purpose** |
| --- | --- | --- | --- | --- |
| CN_01230_qRT_F(3) | GTCAGACGACACCGATGACTGG | | qRT-PCR for MP98 expression analysis | |
| CN_01230_qRT_R(3) | GGTTAAGAACAACGGGGCCGTG | | qRT-PCR for MP98 expression analysis | |
| CN_01239_qRT_F(2) | TGGTGGGAATACGTACGGAGCC | | qRT-PCR for MP84 expression analysis | |
| CN_01239_qRT_R(2)  Cn_01239_T_F_EcoRⅤ  Cn_01239_T_B_NotⅠ  Cn_01239_O_F_Xho  Cn_01239_O_B_EcoRⅤ  CN_01239_sq_F | GACGGAAACATGTCCCTGAGCG  GCGCGATATCTAGATGCCATGTGCACTT  AGGCGGCCGCAAGGCTTCGCCAGAACAGAT  GCGCCTCGAGCCAACTCCAATGGTCAGT  GCGCGATATCCTAGTGGTGGTGGTGGTGGTGAGAAGACGCTTCGGAGCT  GAACGGCCCGTTAGAATG | | qRT-PCR for MP84 expression analysis  Amplification of MP84 terminator  Amplification of MP84 terminator  Amplification of His-tagged MP84 ORF  Amplification of His-tagged MP84 ORF  Confirmation of His-tagged MP848 ORF | |
| N del MP84-1 | CGAATTGGGTACCGGGCC | | Construction of MP84 lacking *N*-glycosylation sites | |
| N del MP84-2 | ACTCCGGCTGTCAGCACC | | Construction of MP84 lacking *N*-glycosylation sites | |
| N del MP84-3 | GGTGCTGACAGCCGGAGT | | Construction of MP84 lacking *N*-glycosylation sites | |
| N del MP84-4 | GCTCAGAACGCCATATCGTC | | Construction of MP84 lacking *N*-glycosylation sites | |
| N del MP84-5 | GACGATATGGCGTTCTGAGC | | Construction of MP84 lacking *N*-glycosylation sites | |
| N del MP84-6 | GCCTCAACGCTTGCAACAG | | Construction of MP84 lacking *N*-glycosylation sites | |
| N del MP84-7 | GGCATCTAGATATCCTAGTGGTG | | Construction of MP84 lacking *N*-glycosylation sites | |
| H3 promoter_F | GTACCGGGCCCCCCCTCGAGCGGCAGATACGATATGTTGGC | | Infusion of the H3 promoter with MP84 ORF in pJAFS1 vector | |
| Infusion MP84_R | AAAAGTGCACATGGCATCTAGATATCCTAGTGGTGGTGGTG | | Infusion of the H3 promoter with MP84 ORF in pJAFS1 vector | |
| H3P+MP84ORF confirm | GCGTATCAGGTTGGAACTGG | | Confirmation of the H3 promoter-Cn01239N(61, 149, 279, 293)A-His insertion | |
| CN_1239_O_B_ EcoRV | AGCTCCGAAGCGTCTTCTCACCACCACCACCACCACTAGGATATCTAGA | | Confirmation of the H3 promoter-Cn01239N(61, 149, 279, 293)A-His insertion | |

**Supplementary Figures**

**Fig. S1**


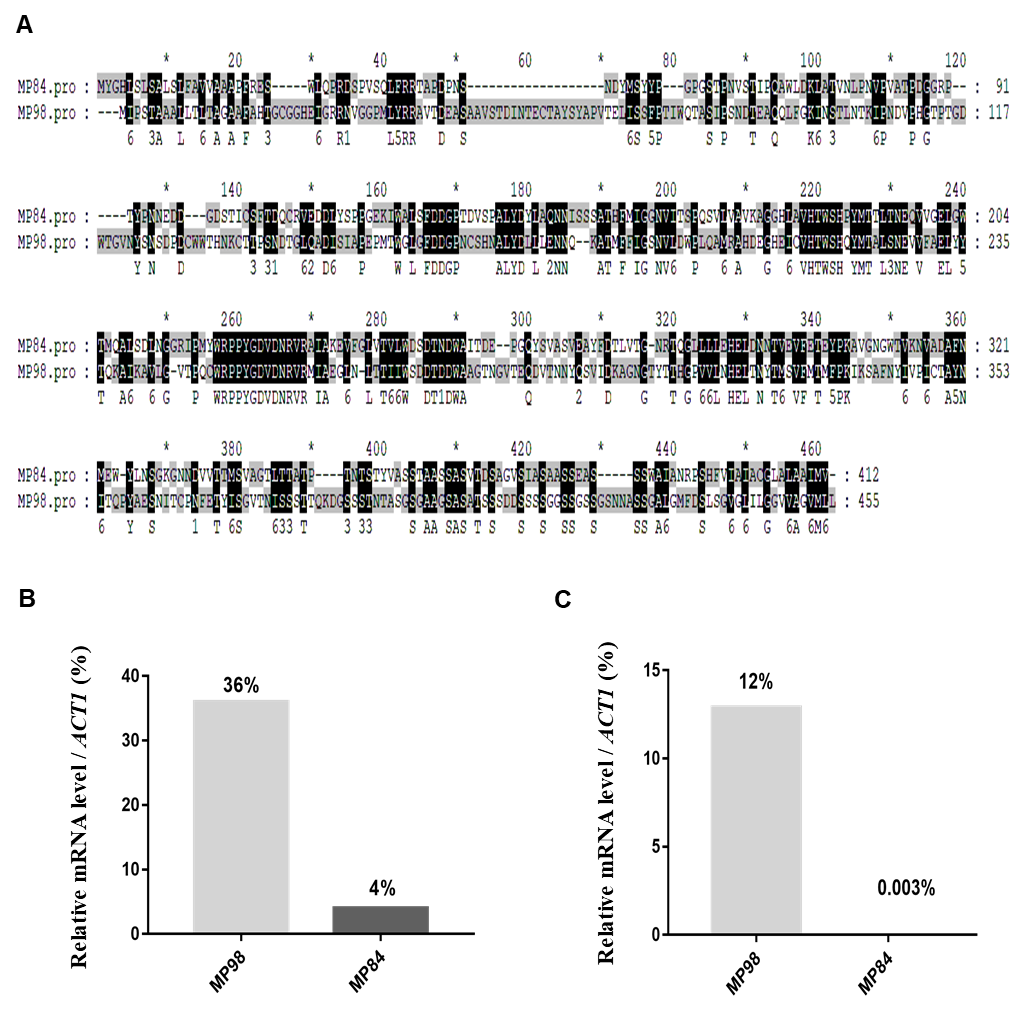


**Supplementary Figure 1. Comparison of amino acid sequences and transcript levels between MP98 and MP84.** (A) Alignment of the amino acid sequences of MP98 and MP84. (B) Relative expression levels of MP98 and MP84 based on the previous RNA-Seq analysis data of *C. neoformans* H99 strain cultivated in a YPD medium (Cheon *et al*., 2011). (C) qRT-PCR analysis of the mRNAs of MP98 and MP84. Total RNAs were prepared from *Cryptococcus* cells at the exponential phase and synthesized as cDNAs, which were used as a template for qRT-PCR with gene-specific primers (Table S1). Relative mRNA levels were represented by the actin gene as a control.

**Fig. S2**

**
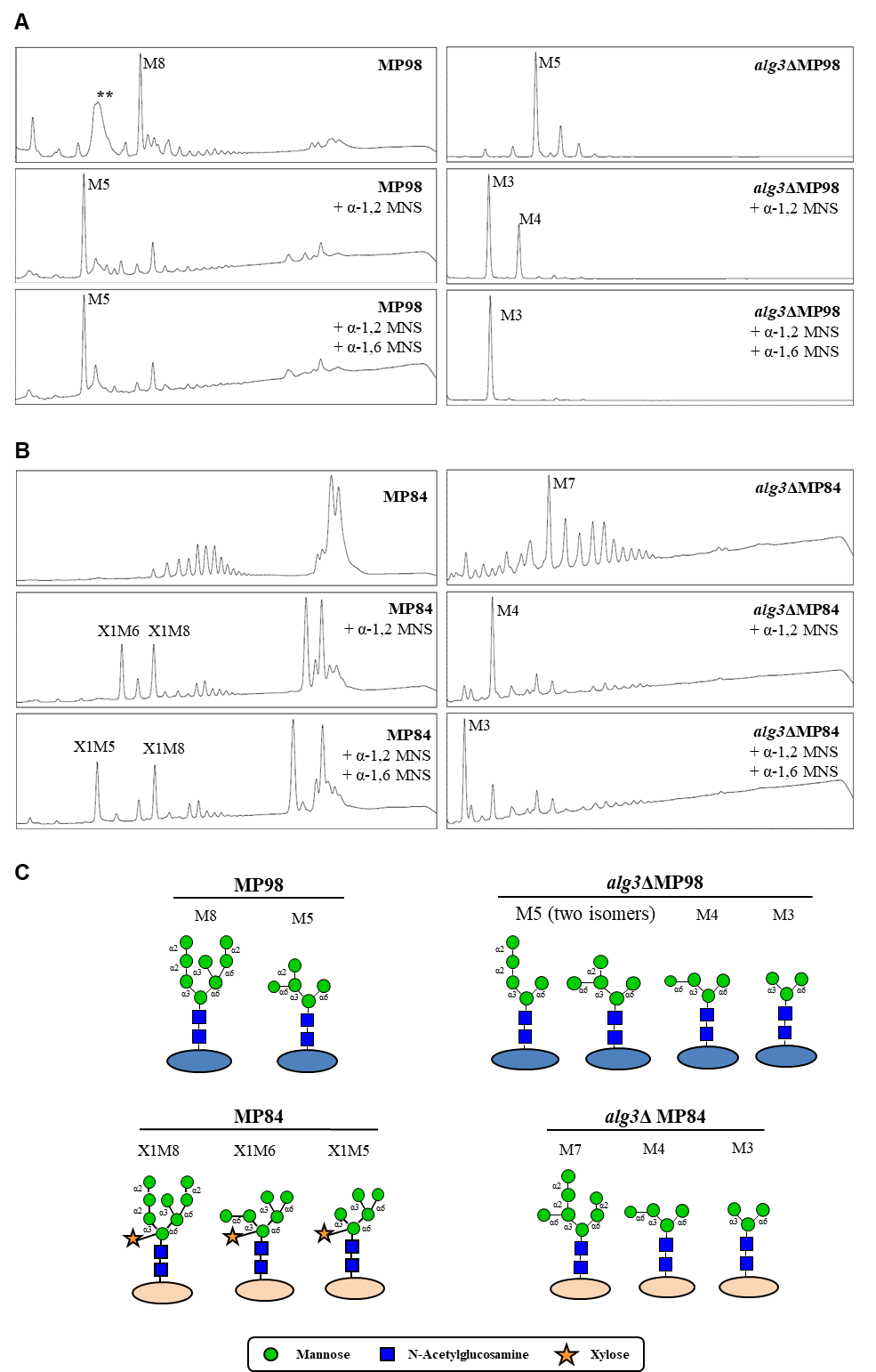
**

**Supplementary Figure 2. Structural analysis of mannosidase-treated *N*-glycans attached to MPs.** AA-labeled *N*-glycans were treated sequentially with α-1,2 mannosidase (GLYKO) and α-1,6 mannosidase (NEB) at 37°C overnight. After being purified with Microcon-30 centrifugal filters (Millipore), the samples were analyzed via HPLC. (A) Characterization of the *N*-glycan structure of MP98 and *alg3*ΔMP98. **, Unidentified nonspecific peak. (B) Characterization of the *N*-glycan structure of MP84 and *alg3*ΔMP84. (C) Structures of *N*-glycans assembled on MP98 and MP84 secreted from WT and *alg3Δ*. **M** and **X** represent mannose and xylose, respectively.

**Fig. S3**

**
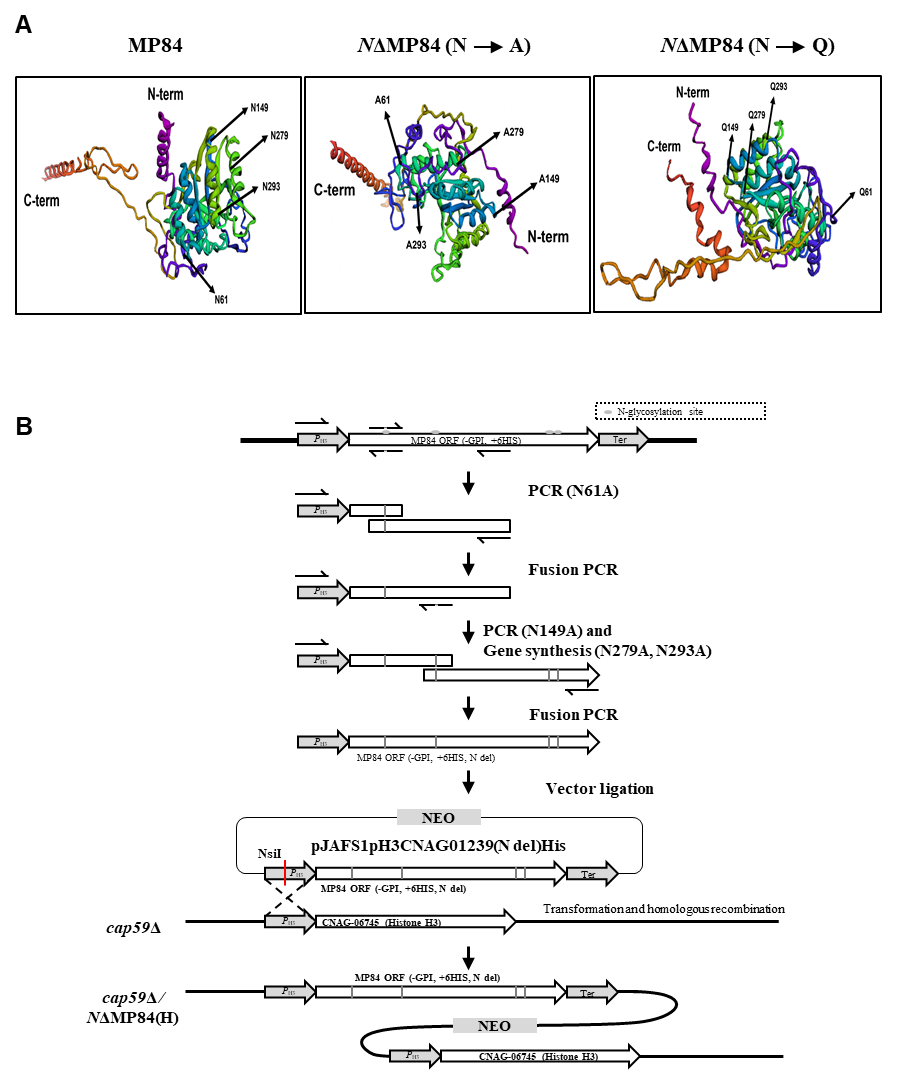
**

**
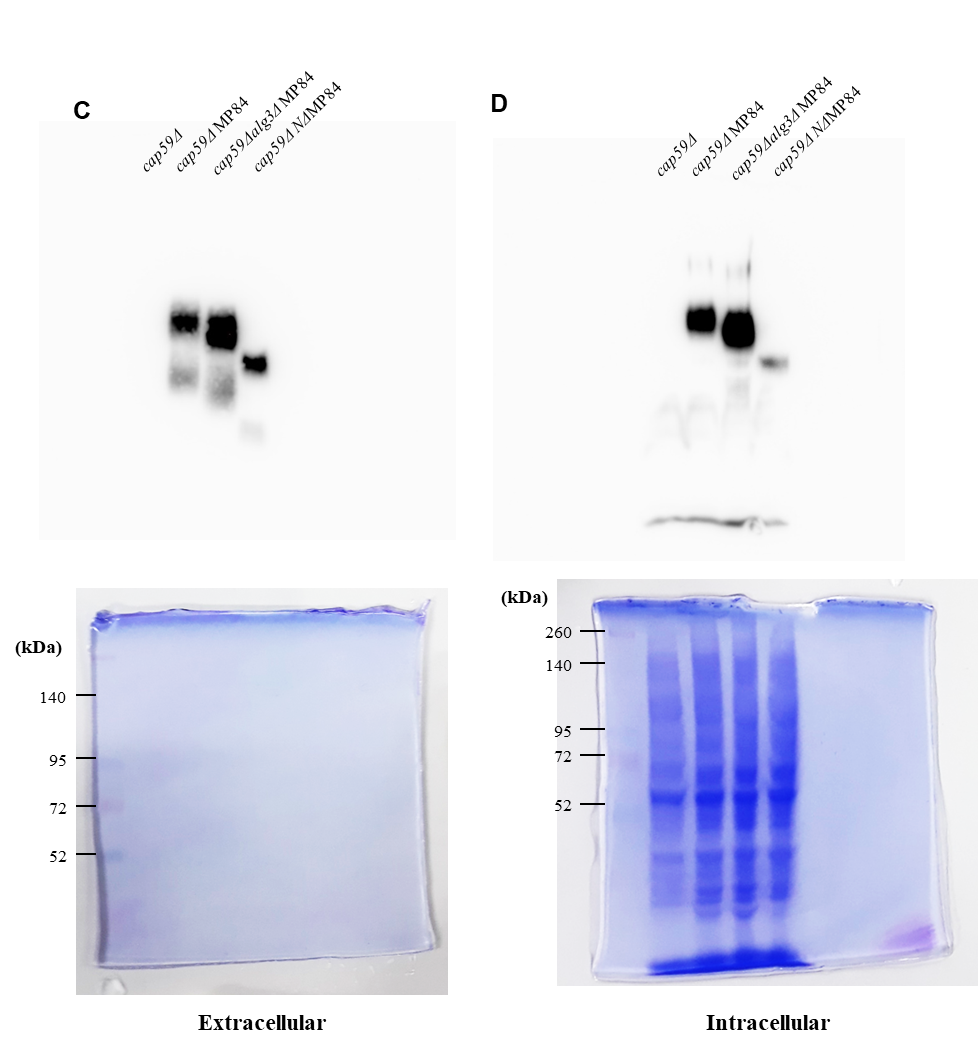
**

**Supplementary Figure 3.** (A) Predicted 3D structures of MP84 and its mutated forms. The asparagine (N) residues in four *N*-glycosylation sites of MP84 were substituted with either alanine (A) or glutamine (Q), and the predicted 3D structures of MP84 variants were analyzed using Robetta (Baker Lab, <http://robetta.bakerlab.org/>). (B) Construction scheme to eliminate four predicted *N*-glycosylation sites in MP84. All asparagine residues at *N*-glycosylation sites were exchanged with alanine residues by protein engineering and chemical gene synthesis. The pJAFS1pH3CNAG01239(N del)His vector expressing MP84 that completely lacked *N*-glycans (*N*ΔMP84) under the control of histone 3 promoter (*P*_H3_) was integrated into the genome of *C. neoformans* WT strain in the background of *cap59*Δ mutation through single homologous recombination. (C) Western blot analysis of culture supernatants (extracellular fraction) to detect the His-tagged MP84 secreted by the WT, *alg3*Δ strains and His-tagged *N*ΔMP84 secreted by WT. (D) Western blot analysis of soluble cell lysates (intracellular fraction) to detect the His-tagged MP84 expressed in the WT, *alg3*Δ strains and His-tagged *N*ΔMP84 expressed in WT.

**Fig. S4**

**
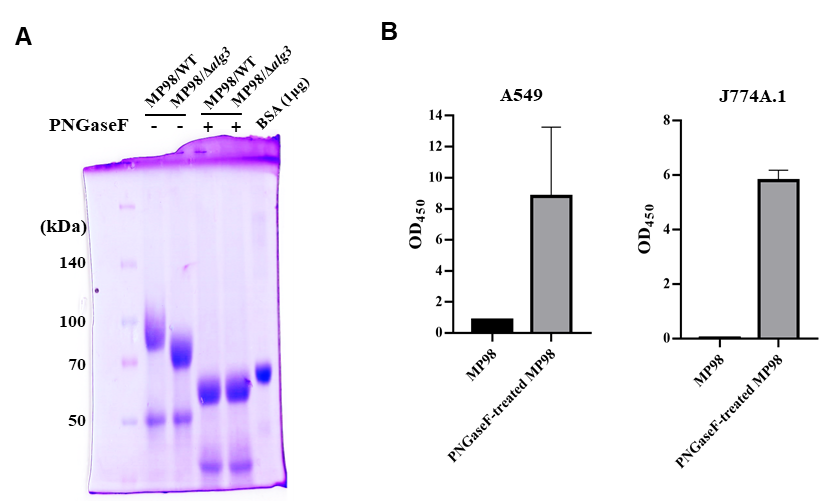
**

**Supplementary Figure 4.** (A) Treatment of MP98 with PNGase F. PNGase F was applied to the purified *alg3*ΔMP98 protein to obtain MP98 lacking *N*-glycans. The *N*-glycan-removed MP98 was subsequently repurified using Ni-Sepharose beads to eliminate PNGase F. (B) Comparison of cell adhesion activities between the MP98 protein carrying the WT *N*-glycans and the PNGase F-treated MP98 protein.

**Fig. S5**

**
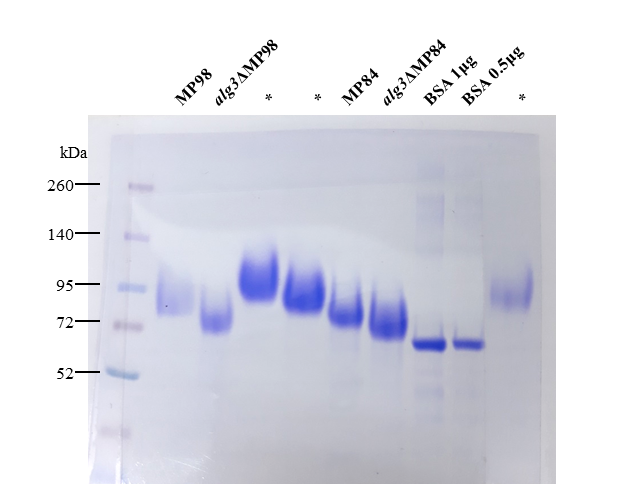
**

**Supplementary Figure 5.** Raw data of Coomassie Blue staining of purified MPs from *C. neoformans* WT and *alg3*Δ strains. *, *C. neoformans* MP88 proteins purified from *C. neoformans* WT and *alg3*Δ strains, which were not reported in this study.  **Fig. S6**

**
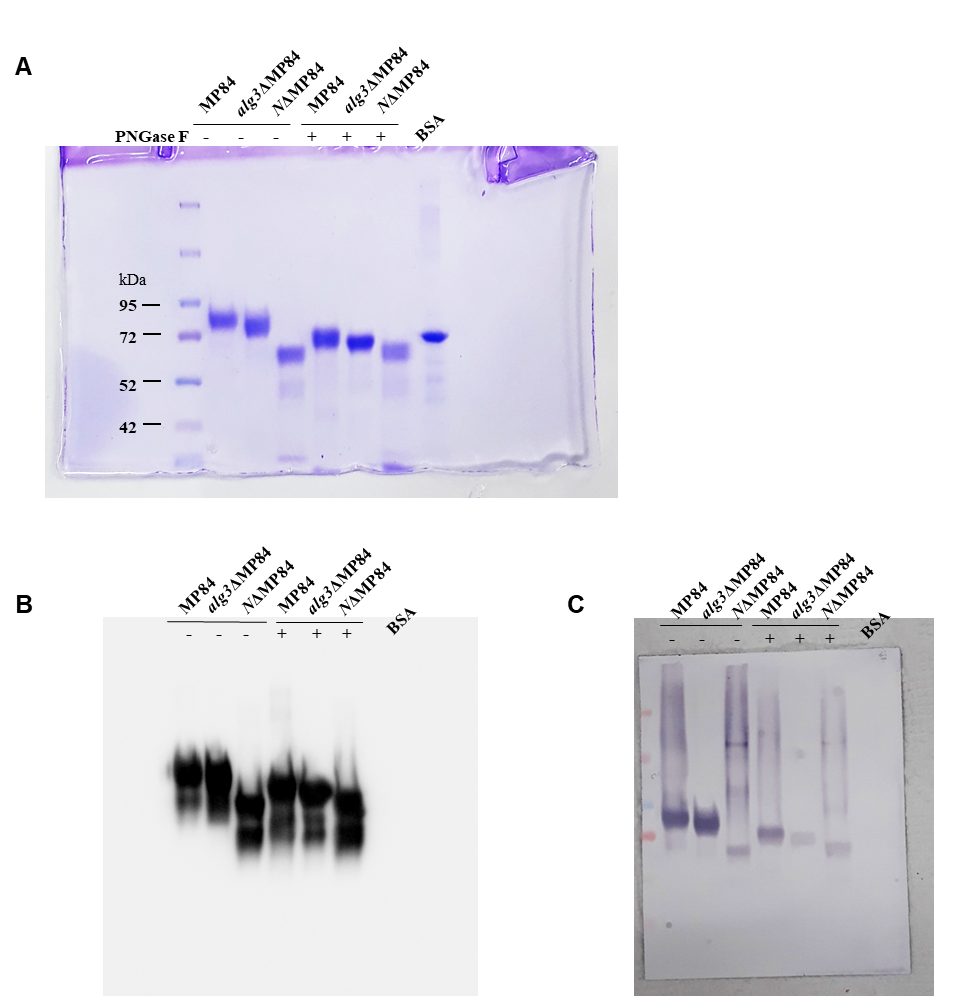
**

**Supplementary Figure 6.** Raw data of SDS-PAGE, Western blot analysis and lectin-blotting of recombinant MP84s with and without PNGase F treatment. (A) SDS-PAGE of MP84 secreted by the WT and *alg3*Δ strains, and the MP84 lacking *N-*glycosylation sites. (B) Western blot analysis of various MP84s using anti-His antibody. (C) Lectin-blotting (GNA-AP) of various MP84s.

**Supplementary References**

1. Cheon, S. A. *et al.* Unique evolution of the UPR pathway with a novel bZIP transcription factor, Hxl1, for controlling pathogenicity of *Cryptococcus neoformans*. *PLOS Pathogens* **7**, e1002177 (2011).
2. Park, J. N., *et al*. Unraveling unique structure and biosynthesis pathway of *N*-linked glycans in human fungal pathogen *Cryptococcus neoformans* by glycomics analysis. *J. Biol. Chem.* **287**, 19501-19515 (2012).
3. Thak, E. J. *et al.* Core *N*-glycan structures are critical for the pathogenicity of *Cryptococcus neoformans* by modulating host cell death. *mBio* **11**, e00711-00720 (2020).
